# Explainable artificial intelligence for genotype-to-phenotype prediction in plant breeding: a case study with a dataset from an almond germplasm collection

**DOI:** 10.3389/fpls.2024.1434229

**Published:** 2024-09-09

**Authors:** Pierfrancesco Novielli, Donato Romano, Stefano Pavan, Pasquale Losciale, Anna Maria Stellacci, Domenico Diacono, Roberto Bellotti, Sabina Tangaro

**Affiliations:** ^1^ Dipartimento di Scienze del Suolo, della Pianta e degli Alimenti, Università degli Studi di Bari Aldo Moro, Bari, Italy; ^2^ Istituto Nazionale di Fisica Nucleare, Sezione di Bari, Bari, Italy; ^3^ Dipartimento Interateneo di Fisica “M. Merlin”, Università degli Studi di Bari Aldo Moro, Bari, Italy

**Keywords:** genotype-phenotype prediction, machine learning, explainable artificial intelligence, shelling fraction, almond

## Abstract

**Background:**

Advances in DNA sequencing revolutionized plant genomics and significantly contributed to the study of genetic diversity. However, predicting phenotypes from genomic data remains a challenge, particularly in the context of plant breeding. Despite significant progress, accurately predicting phenotypes from high-dimensional genomic data remains a challenge, particularly in identifying the key genetic factors influencing these predictions. This study aims to bridge this gap by integrating explainable artificial intelligence (XAI) techniques with advanced machine learning models. This approach is intended to enhance both the predictive accuracy and interpretability of genotype-to-phenotype models, thereby improving their reliability and supporting more informed breeding decisions.

**Results:**

This study compares several ML methods for genotype-to-phenotype prediction, using data available from an almond germplasm collection. After preprocessing and feature selection, regression models are employed to predict almond shelling fraction. Best predictions were obtained by the Random Forest method (correlation = 0.727 ± 0.020, an *R* ^2^ = 0.511 ± 0.025, and an RMSE = 7.746 ± 0.199). Notably, the application of the SHAP (SHapley Additive exPlanations) values algorithm to explain the results highlighted several genomic regions associated with the trait, including one, having the highest feature importance, located in a gene potentially involved in seed development.

**Conclusions:**

Employing explainable artificial intelligence algorithms enhances model interpretability, identifying genetic polymorphisms associated with the shelling percentage. These findings underscore XAI’s efficacy in predicting phenotypic traits from genomic data, highlighting its significance in optimizing crop production for sustainable agriculture.

## 1 Background

Next generation DNA sequencing technologies nowadays allow the cost-effective identification and call of a large number of single nucleotide polymorphisms (SNPs), using whole genome resequencing (WGS) and reduced representation sequencing (RRS) approaches Pavan et al. (2020). In turn, this facilitates the prediction of phenotypes based on genomic data, using genomic selection (GS) methods. For both annual and perennial crops, GS has the potential to dramatically reduce the time and the cost required for the development of new cultivars Crossa et al. (2017). The advantage of GS is even more noticeable for fruit tree species, for which phenotypic selection requires to grow plants for several years until the completion of the juvenile period. However, despite these remarkable strides, the accurate prediction of phenotypes from genomic data remains an enduring challenge in the field of plant breeding van Dijk et al. (2021). In the contemporary landscape of “big data” available for crop species, the ability predict phenotypes from genotypic information holds paramount importance, particularly in the context of breeding applications. The comprehension of the dynamic interplay between genotypic variation and resulting phenotypes offers profound insights into fundamental aspects of plant physiology and development Tong and Nikoloski (2021).

While traditional linear regression models have been valuable tools in genetic studies, they may have limitations in capturing the nuanced relationships between genotypes and phenotypes. These models often assume linearity, which may not hold true for complex biological interactions. Additionally, they may struggle with high-dimensional genomic data, leading to issues such as overfitting and multicollinearity, which can reduce predictive accuracy and reliability Guzzetta et al. (2010); Okser et al. (2014); Danilevicz et al. (2022). However, the emergence of Machine Learning (ML) techniques, notably non-linear models and tree-based models, has heralded a paradigm shift in this domain Li et al. (2018); Abdollahi-Arpanahi et al. (2020); Wang et al. (2022); Azodi et al. (2019). These sophisticated methodologies excel at generating precise predictions from the extensive biological datasets generated in plant genotyping and phenotyping studies John et al. (2022); Sehrawat et al. (2023). ML, as a computational approach for discerning predictive patterns within data, holds significant promise in revolutionizing genotype-to-phenotype predictions in plant science Guo and Li (2023); Wang et al. (2023). ML techniques have become essential tools for plant researchers, facilitating the processing and integration of vast datasets to unveil deeper insights into the intricate relationships between genotypes and phenotypes. Recent reviews and studies highlight the application and comparison of various ML models in genomic prediction, showcasing their effectiveness in different contexts Chafai et al. (2023); Lourenço et al. (2024). Moreover, recent advancements in machine learning have led to the development of explainable artificial intelligence (XAI) algorithms, aimed at elucidating the inner workings of machine learning models often deemed as “black boxes” Novielli et al. (2024); Linheiro et al. (2023); Mostafa et al. (2023); Van Stein et al. (2022); Cilli et al. (2022); Lombardi et al. (2022). These XAI algorithms enhance the reliability and interpretability of results by elucidating the variables that have the most significant impact on the predictive outcome. The connection between prediction accuracy and interpretability is crucial in breeding applications, as understanding the genetic mechanisms underlying trait predictions can inform better breeding decisions. For example, recent studies have applied explainable AI to genomic prediction in crops, demonstrating the value of this approach in identifying marker effects and estimating heritability Coelho de Sousa et al. (2022); Sousa et al. (2020). This development is particularly promising in the context of crop breeding, as it enables the identification of key SNPs driving the regression model, potentially leading to significant breakthroughs in predicting phenotypic traits such as yield.

Despite the advances in genomic selection, there remains a significant gap in accurately predicting phenotypes from high-dimensional genomic data, particularly in identifying the key genetic factors that most impact these predictions. This study aims to address these challenges by leveraging XAI techniques in conjunction with advanced machine learning models to enhance the predictive accuracy of genotype-to-phenotype predictions. Our specific research goals are to demonstrate the effectiveness of XAI in identifying key genomic regions associated with phenotypic traits. We hypothesize that advanced machine learning models, particularly tree-based methods, will outperform traditional linear models in predicting phenotypic traits from genomic data. Additionally, we expect that the use of XAI will reveal significant SNPs and genomic regions that are strongly associated with phenotypic traits, such as shelling fraction.

## 2 Materials and methods

### 2.1 Phenotipic and genotipic data

Our study aimed to compare three machine learning methods to investigate the relationship between plant genetic data and phenotypic traits. To accomplish this, we utilized genotypic and phenotypic data from 98 cultivars from the CREA-AA (Italian Council for Agricultural Research and Analysis of Agricultural Economics—Section Agriculture and Environment) *ex situ* germplasm collection, previously described by Pavan et al. (2021); Pavan et al. (2021). Almond, as one of the primary tree nut species worldwide and among the oldest domesticated tree species, has its genome organized into eight chromosomes Delplancke et al. (2013).

The dataset comprised 98 individuals, each represented by a unique cultivar genotyped. SNP data were obtained by the genotyping-by-sequencing (GBS) RRS approach Elshire et al. (2011), using the Lauranne genome for the alignment of reads Sánchez-Pérez et al. (2019). Four-year data on kernel and fruit weight were used to calculate the average shelling fraction (i.e. the ratio of kernel weight to total fruit weight), which was further considered as target phenotypic variable. Detailed information about the cultivars and the phenotypic variable can be found in **Supplementary Table 1**. This variable is very important for the technological quality of the product and it is strongly linked to the genotype rather than the agronomic practices The International Union for the Protection of New Varieties of Plants-UPOV (https://www.upov.int/edocs/mdocs/upov/en/tc_edc/2011/tg_56_4_proj_3_e.pdf). Data were preprocessed to ensure quality control using TASSEL v.556. Marker quality control involved filtering for biallelic SNP loci with a minor allele frequency > 0.05 and a call rate > 0.7, resulting in 93119 single-nucleotide polymorphisms (SNPs) available for analysis. Subsequently, Linkage Disequilibrium (LD) pruning was conducted using the LD pruning algorithm in PLINK v.1.90 Ye et al. (2019); Nimmakayala et al. (2014). This algorithm calculates pairwise *R* ^2^ for all marker pairs in sliding windows with a size of 50 markers and an increment of 5 markers, removing the first marker of pairs in which *R* ^2^< 0.5.

The Variant Call Format (VCF) file containing the SNPs underwent additional encoding to prepare it for the subsequent machine learning framework phase: homozygous variants, indicated by 0/0, were encoded as 0; heterozygous variants, indicated by 0/1 and 1/0, were encoded as 1; and homozygous variants, indicated by 1/1, were encoded as 2. Here, “0” denotes the reference allele, and “1” denotes the alternative allele.

### 2.2 Workflow analysis

**Figure 1:**
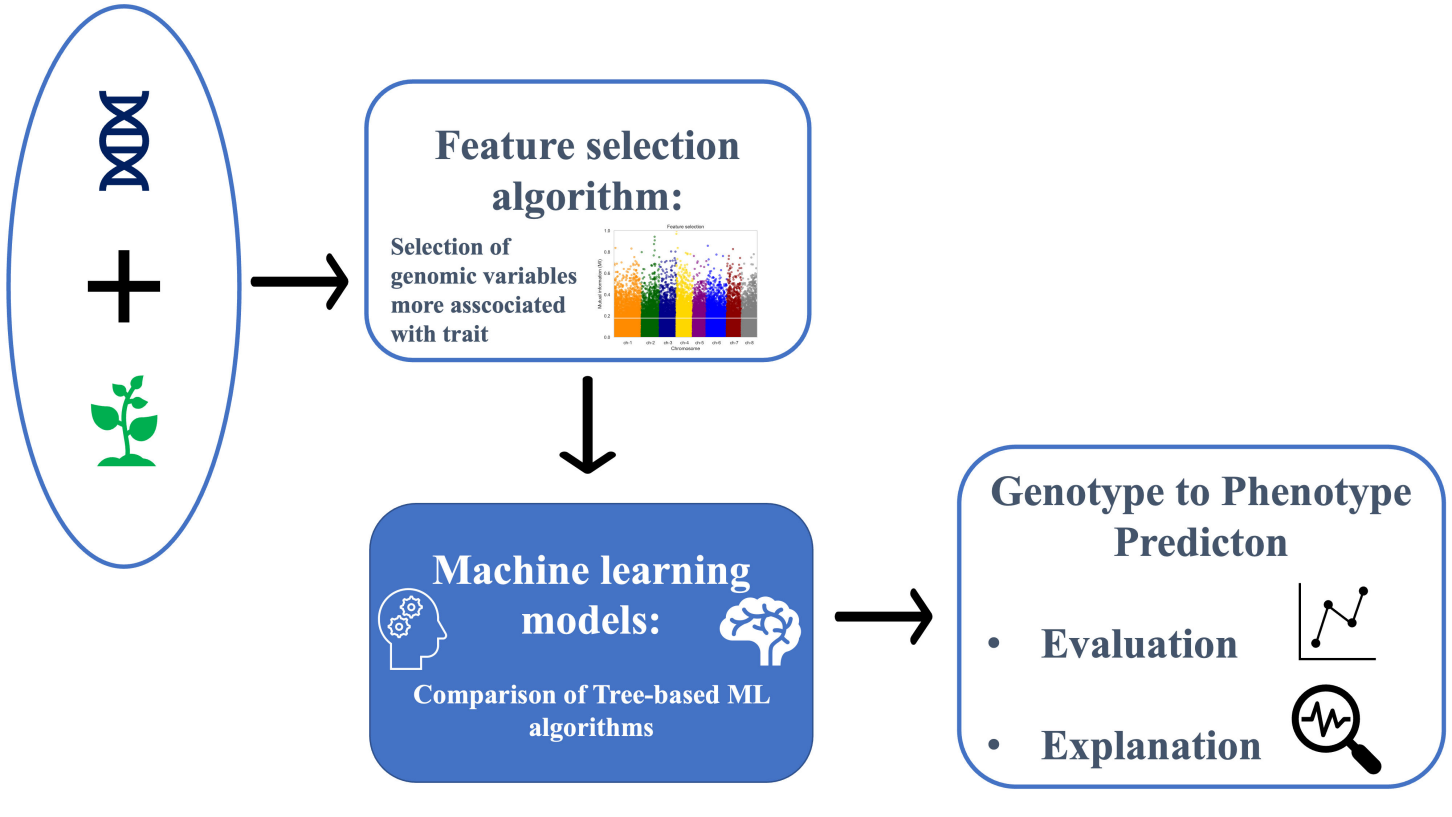
Flowchart illustrating the analysis workflow. Input genetic data underwent preprocessing steps before being subjected to a feature selection algorithm. Subsequently, the preprocessed data were fed into tree-based machine learning algorithms to evaluate regression results and provide explanations.

**Figure 1** illustrates the general schema followed to conduct the analysis. After preparation, SNP and phenotypic data were input into the ML framework. Due to the limited number of plants available for study compared to the number of SNP variables, to avoid the curse of dimensionality Crespo Márquez (2022); Altman and Krzywinski (2018), a feature selection algorithm was adopted. The curse of dimensionality refers to various phenomena that arise when analyzing and organizing data in high-dimensional spaces. In our study, having a high number of SNPs relative to the number of plants can lead to overfitting and poor model generalization because the model may fit the noise in the data rather than the actual signal. Subsequently, three tree-based ML regression models and traditional regression models (e.g., gBLUP and rrBLUP) were compared, and their performance was evaluated using different evaluation metrics (Pearson correlation, *R* ^2^, and RMSE). The SHAP (SHapley Additive exPlanations) values algorithm was utilized to provide an interpretable explanation for the model’s predictions. This method helps to identify and quantify the contribution of each SNP to the predicted phenotypic traits, ensuring transparency and understanding of the model’s decision-making process. To validate the procedure, 10-fold cross-validation (CV) was employed. Feature selection was nested within the cross-validation to prevent data leakage. Data leakage occurs when information from outside the training dataset is used to create the model, leading to overly optimistic performance estimates. When performing feature selection on all of the data and then cross-validating, the test data in each fold of the CV procedure could be used to choose the features, which biases the performance analysis (Samala et al. (2020); Saravanan et al., 2018). Thus, feature selection was applied to each split of the CV, followed by training an ML regression model. Moreover, the cross-validation procedure was repeated 15 times to ensure robustness in the analysis, with each repetition involving different splits of the folds. After each repetition of the cross-validation, the model assessment metric was computed, providing uncertainty associated with the results to obtain a statistical analysis of the findings.

The sequence of steps involved in the analysis are summarized in **Table 1**.

**Table 1:** Modelling strategy steps and cross-validation details.

Step	Description	Validation
**[S1] Data Preparation**	SNP and phenotypic data were prepared for input into the Machine Learning (ML) framework. The SNP data underwent quality control filtering, including marker quality control (MAF, call rate) and Linkage Disequilibrium (LD) pruning.	
**[S2] Feature Selection**	Due to the limited number of plants compared to the number of SNPs, a feature selection algorithm was applied to mitigate the curse of dimensionality. Mutual information was used to identify and retain the most informative SNPs.	Steps S2 to S4 were done by employing 10-fold cross-validation (CV). The procedure was repeated 15 times to ensure robustness, with each repetition involving different splits. After each repetition, the model assessment metric was computed, providing uncertainty associated with the results for statistical analysis.
**[S3] Model** **Comparison**	Three tree-based ML regression models (Random Forests, Adaboost, and Gradient Boosting) and traditional regression models (e.g., gBLUP and rrBLUP) were compared. Their performances were evaluated using Pearson correlation, R², and RMSE.	
**[S4] Interpretability**	The SHAP (SHapley Additive exPlanations) algorithm was used to provide explanations for the model results. This method helps identify and quantify the contribution of each SNP to the predicted phenotypic traits.	

### 2.3 Feature selection

Feature selection is a critical step in data preprocessing, aiming to identify and retain the most informative features while discarding irrelevant ones, thereby enhancing the model’s performance Ross (2014); Kraskov et al. (2004); Tangaro et al. (2015). In our study, we employed a feature selection technique based on Mutual Information (MI) Gain. This method, a univariate filtering approach, calculates the mutual information for continuous target variables in regression problems, relying on the entropy of the variables Guo et al. (2020).

Mutual Information quantifies the dependency between variables, where higher values indicate stronger dependency. It essentially measures the amount of information one variable provides about another. Like other feature selection techniques, the goal of MI Gain is to reduce the size of the input feature set. This reduction can simplify the problem, decrease computational time, and potentially improve model performance.

In our approach, SNPs are ranked based on their MI scores, and those above the 80th percentile of the MI scores are selected for further analysis. This percentile-based threshold ensures that we retain the most informative SNPs, focusing on the top 20% that provide the highest dependency information.

### 2.4 Benchmark methods

To provide a comprehensive comparison, we included traditional genomic regression methods as benchmarks. Specifically, we performed genomic best linear unbiased prediction (GBLUP) and ridge regression best linear unbiased prediction (rrBLUP) as benchmark methods Crossa et al. (2017). These models do not involve variable selection and serve as a reference for evaluating the performance of the machine learning models with feature selection.

GBLUP and rrBLUP are widely used in genomic prediction due to their simplicity and robustness. GBLUP uses a mixed linear model approach that incorporates all available SNPs as random effects, assuming a common variance for all SNPs. rrBLUP is a variant of GBLUP that applies ridge regression to handle multicollinearity among SNPs, thus providing stable and reliable predictions Clark and van der Werf (2013); Tan et al. (2017); Nazzicari and Biscarini (2022).

### 2.5 Tree-based ML regressors

The machine learning models chosen for regression are tree-based ML models, which typically perform effectively on tabular data Grinsztajn et al. (2022); Manduchi et al. (2021). The models selected are AdaBoost, RandomForest, and Gradient Boosting.

**AdaBoost:** The core principle of AdaBoost is to fit a sequence of weak learners, such as small decision trees, on repeatedly modified versions of the data Freund and Schapire (1997). The predictions from all weak learners are then combined to produce the final prediction. At each boosting iteration, the data modifications involve adjusting weights assigned to each training sample based on prediction accuracy.

**Gradient Boosting:** Gradient boosting regression tree algorithms utilize an ensemble learning technique, amalgamating multiple individual regression trees, also known as weak learners, to construct robust forecasting models. This algorithm effectively reduces the error rate associated with weakly learned models, characterized by high bias, low variance, and minimal regularization, thereby enhancing their predictive performance. Boosting algorithms typically comprise three key components: an additive model, weak learners, and a loss function. In the case of gradient boosting machines, the approach involves identifying the deficiencies of weak models by leveraging gradients. Through an iterative process, the algorithm progressively addresses these limitations by iteratively combining base learners to minimize prediction errors. This integration is achieved by employing an additive model while simultaneously minimizing the loss function using gradient descent techniques Friedman (2001).

**Random Forests:** In random forests Breiman (2001), each tree in the ensemble is built from a sample drawn with replacement from the training set. When splitting each node during tree construction, the best split is found through an exhaustive search of feature values from either all input features or a random subset. This randomness decreases the variance of the forest estimator, reducing overfitting. Random forests achieve reduced variance by combining diverse trees, sometimes at the cost of a slight increase in bias.

Hyperparameters play a crucial role in controlling the complexity of the models, avoiding overfitting, and achieving better performance. The hyperparameters varied for the tree-based models used in this study include:

• **Learning Rate** (*learning_rate*): This parameter controls the contribution of each weak learner to the final model in boosting algorithms. A smaller learning rate makes the model more robust to overfitting but requires more trees to achieve optimal performance.

• **Number of Estimators** (*n_estimators*): This parameter specifies the number of weak learners (trees) to be used in the ensemble. Increasing the number of estimators generally improves the model’s performance but also increases computational cost.

• **Maximum Depth** (*max_depth*): This parameter controls the maximum depth of the trees. Limiting the depth of the trees helps to prevent overfitting by ensuring the trees do not become too complex.

To determine the optimal performance of regression in the cross-validation (CV) mode, the following parameters for Gradient Boosting and AdaBoost were varied:

- *learning_rate* ∈ {0.01, 0.05, 0.1, 0.2}
- *n_estimators* ∈ {30, 50, 100, 500}

For Random Forests, the following parameters were varied:

- *max_depth* ∈ {4, 7, 10}
- *n_estimators* ∈ {30, 50, 100, 500}

Hyperparameter tuning was performed using the RandomizedSearchCV Python library to find the best combination of hyperparameters for each model. The optimal hyperparameters identified were as follows:

• **Random Forest:**

 • n_estimators : 500

 • max_depth : 7

• **Gradient Boosting:**

 • n_estimators : 100

 • learning_rate : 0.1

• **AdaBoost:**

 • n_estimators : 500

 • learning_rate : 0.05

To assess the regression results and compare different algorithms, the following metrics were used:

• Pearson correlation:


(1) $$corr=\frac{\sum_{i=1}^{n}(y_{i}-\bar{y})\left({\hat{y}}_{i}-\bar{\hat{y}}\right)}{\sqrt{\sum_{i=1}^{n}{\left(y_{i}-\bar{y}\right)}^{2}\sum_{i=1}^{n}{\left({\hat{y}}_{i}-\bar{\hat{y}}\right)}^{2}}}$$

• Coefficient of determination:


(2) $$R^{2}=1-\frac{\sum_{i=1}^{n}{\left(y_{i}-{\hat{y}}_{i}\right)}^{2}}{\sum_{i=1}^{n}{\left(y_{i}-\bar{y}\right)}^{2}}$$

• Root mean squared error:


(3) $$RMSE=\sqrt{\frac{1}{n}\sum_{i=1}^{n}{\left(y_{i}-{\hat{y}}_{i}\right)}^{2}}$$

where ${\hat{y}}_{i}$ represents the predicted values, $\bar{\hat{y}}$ denotes their average, $y_{i}$ are the observed values of the phenotypic trait, and $\bar{y}$ denotes its average.

### 2.6 SHAP values

Based on game theory, SHAP values assign an importance score to each feature for a given prediction. A positive SHAP value means that the feature has increased the prediction, while a negative SHAP value means that it has decreased the prediction. The larger the absolute value of the SHAP value, the stronger the feature’s impact on the prediction. One of the main benefits of SHAP values is that they are model-agnostic, meaning they can be used to interpret any machine learning model.

The computation of SHAP values involves evaluating the effect of including or excluding each feature from the model. Imagine we have a set of features used to make a prediction. By calculating the difference in the prediction with and without each feature across all possible subsets of features, we can determine the contribution of each feature. This approach ensures that the feature contributions are fairly distributed. To compute SHAP values, we look at the difference in model output when a specific feature is included versus when it is excluded, across all possible combinations of features. This process can be mathematically represented as follows:


(4) $$SHAP_{j}\left(x\right)=\sum_{F\subseteq S-\left\{j\right\}}\frac{\left|F\right|!\left(\left|S\right|-\left|F\right|-1\right)!}{\left|S\right|!}\left[f_{x}\left(F\cup j\right)-f_{x}\left(F\right)\right]$$

where |F|! represents the permutations of features in the subset F,(|S|−|F|−1)! denotes the permutations of features in the subset S−(F∪{j}),|S|! is the total number of feature permutations, and $f_{x}\left(F\cup j\right)$ and $f_{x}\left(F\right)$ represent the regression score obtained by including and not including the *j*-th feature, respectively Lundberg and Lee (2017).

In simpler terms, this formula aggregates the change in prediction when the feature *j* is added to every possible subset of other features. The aggregation is weighted to ensure fairness, considering all possible orders in which features can be added. To make this concept clearer, let’s consider a simplified example with a dataset containing three features: A, B, and C. To compute the SHAP value for feature A for a specific instance, we would:

1. Calculate the model prediction using all subsets that include A and those that don’t.
2. Measure the difference in predictions for each subset pair (with and without A).
3. Aggregate these differences, applying the weighting formula to ensure a fair contribution.

Details about the software and packages used in all the analysis are provided in the **Supplementary Material**.

## 3 Results

The aim of this work was to explore the application of explainable artificial intelligence (XAI) principles to tree-based machine learning models (Random Forests, AdaBoost, and Gradient Boosting) for genomic prediction using almond germplasm data. Specifically, we aimed to construct regression models with feature selection and calculate SHAP values to provide interpretability for the predictions.

**Figure 2:**
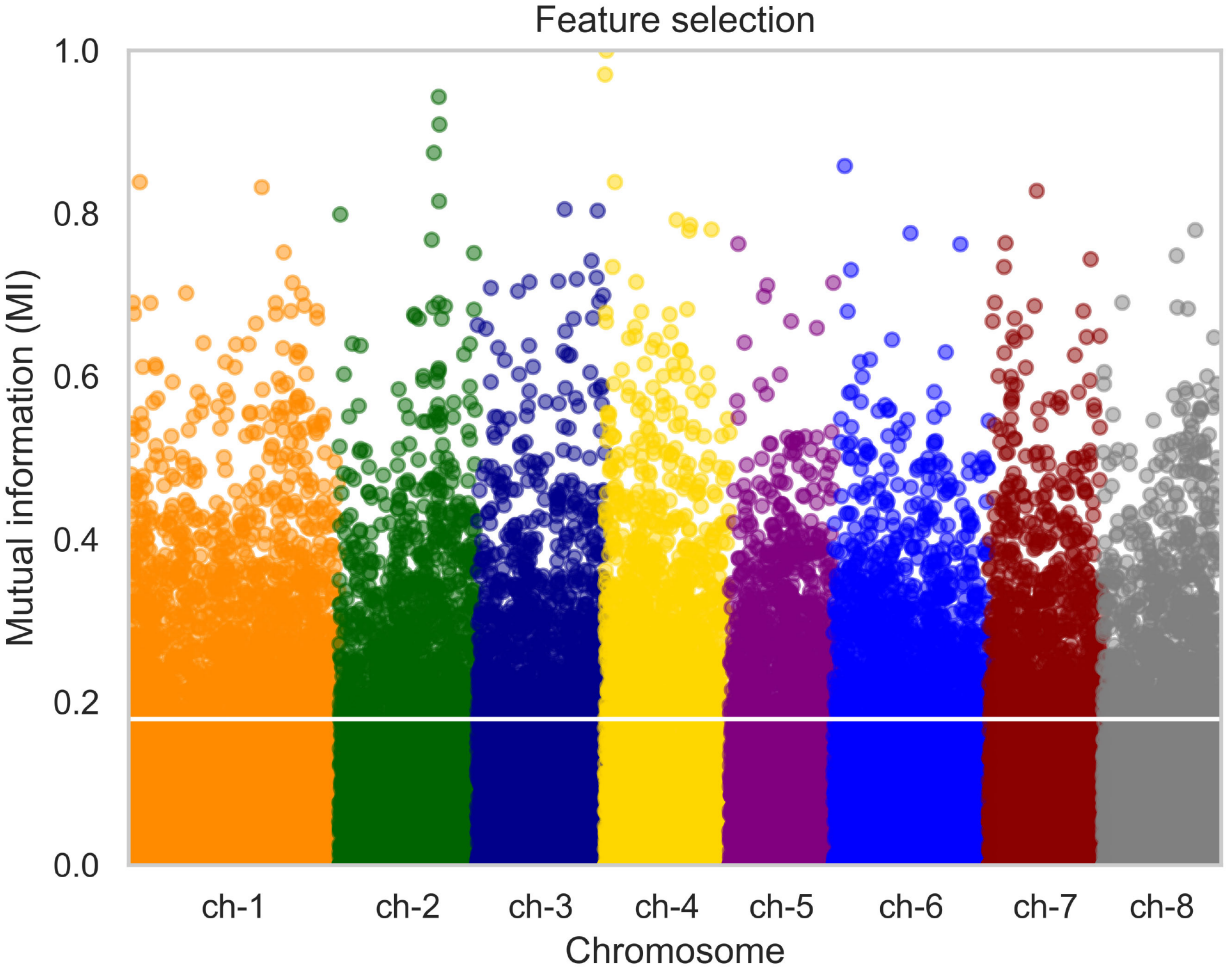
Manhattan plot illustrating the distribution of SNPs for the phenotypic trait of almond shelling fraction. Each point represents a SNP, grouped by chromosome on the x-axis. The y-axis depicts mutual information values. SNPs with mutual information above the 80th percentile were selected for further analysis, as indicated by the horizontal white line. P value < 10^-4^.

The result of data filtering and pruning indicated a substantial reduction in the SNP count from 93119 to approximately 43711. Dimensionality reduction was applied by performing feature selection as described previously. The initial filtering approach served as a quality control measure for SNPs, while LD pruning was used to remove multicollinearity from the SNP data. Subsequently, feature selection was employed to further reduce the number of SNPs, effectively addressing the ‘curse of dimensionality’ and ensuring that only the most informative markers were used in the analysis. For the phenotypic trait of shelling fraction, we analyzed its Manhattan plot, wherein each point denotes a SNP. The x-axis represents the SNPs organized by chromosome, while the y-axis depicts mutual information values. SNPs with mutual information values above the 80th percentile were selected, resulting in the retention of approximately 8600 SNPs. An example of the Manhattan plot corresponding to one fold of the cross-validation is depicted in **Figure 2**. The horizontal white line denotes the threshold corresponding to the 80th percentile.

**Figure 3:**
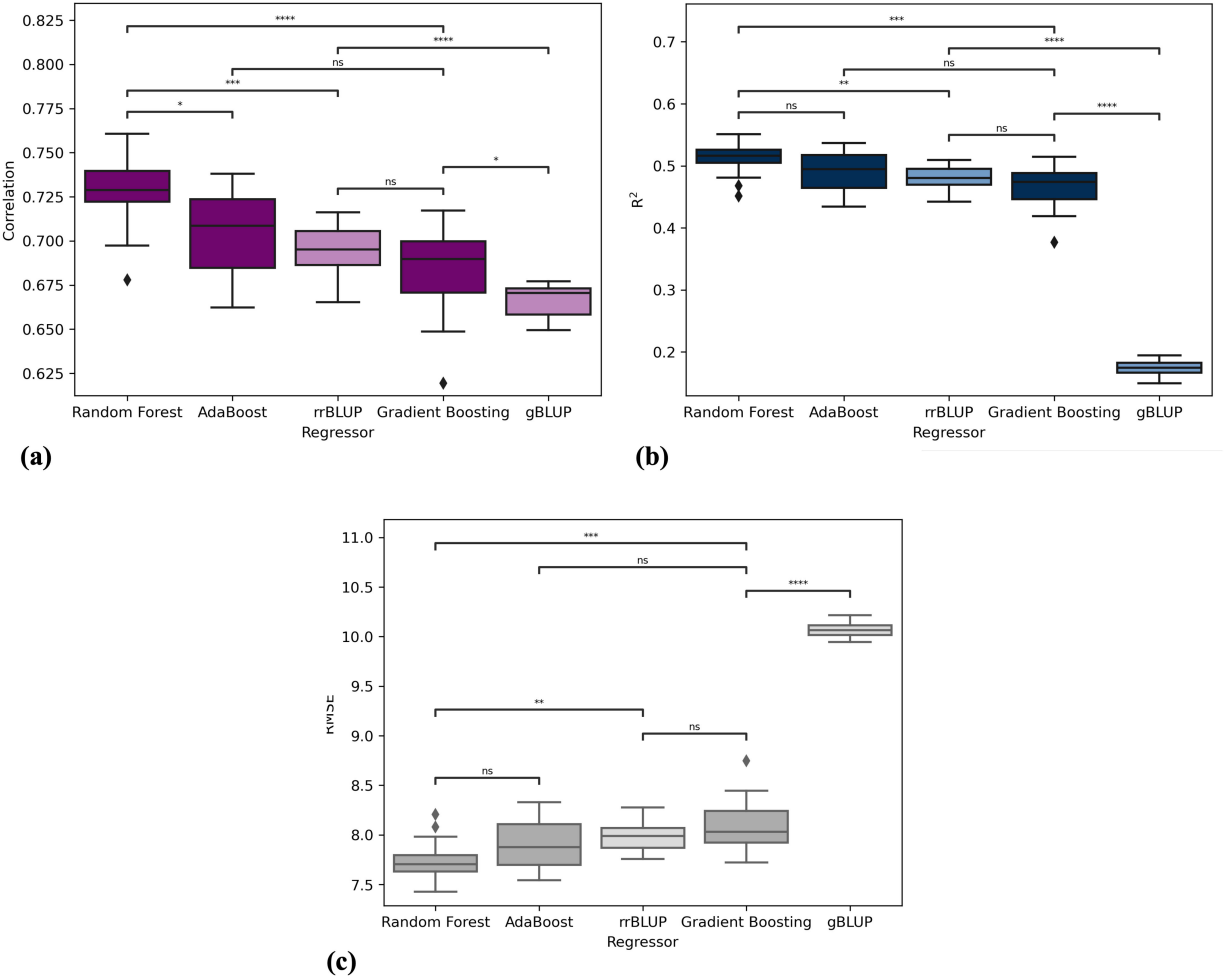
Performance comparison of machine learning algorithms in terms of **(A)** correlation, **(B)** *R* ^2^, and **(C)** RMSE. Each boxplot represents the distribution of scores obtained from 15 repetitions of crossvalidation for the following algorithms: Random Forest, Gradient Boosting, AdaBoost, gBLUP and rrBLUP. Significance stars indicate the results of Mann-Whitney U test comparing the distributions of each algorithm’s performance scores. The significance levels are denoted as follows: ns (not significant), ^∗^ (10^−2^ < *p* − *value <* 5 × 10^−2^), ^∗∗^ (10^−3^ < *p* − *value <* 10^−2^), ^∗∗∗^ (10^−4^ < *p* − *value <* 10^−3^), and **** *p* value < 10^-4^.

**Table 2** presents the results of the five models in terms of correlation, *R* ^2^, and RMSE. The table displays the results obtained by repeating the cross-validation procedure 15 times, presenting the average values along with their standard deviations. **Figure 3** depicts the performance of the three algorithms as boxplots of the distributions obtained in the 15 repetitions of the procedure. **Figures 3A–C** represent correlation, *R* ^2^, and RMSE, respectively. Each boxplot on the x-axis corresponds to one algorithm (Random Forest, Gradient Boosting, and AdaBoost), while the y-axis represents the scores. It can be observed that Random Forest statistically outperforms the other two algorithms in terms of correlation. Regarding *R* ^2^ and RMSE, Random Forest outperforms Gradient Boosting, gBLUP and rrBLUP but has statistically comparable results to AdaBoost. Consequently, Random Forest is considered the best regressor, with average results of correlation of 0.727 ± 0.020, a *R* ^2^ of 0.511 ± 0.025, and an RMSE of 7.746 ± 0.199. XAI results are shown for this algorithm, but the results of the other models are consistent with those of Random Forest. The statistical tests for comparing the distributions were conducted using the Mann-Whitney U test Mann and Whitney (1947).

**Table 2:** Results of regression models in terms of correlation, *R* ^2^, and RMSE.

Regressor	Correlation	*R* ^2^	RMSE
**Random Forest**	**0**.**727** ± **0**.**020**	**0**.**511** ± **0**.**025**	**7**.**746** ± **0**.**199**
Gradient Boosting	0.682 ± 0.025	0.464 ± 0.035	8.106 ± 0.264
AdaBoost	0.703 ± 0.024	0.489 ± 0.031	7.912 ± 0.243
gBLUP	0.666 ± 0.009	0.173 ± 0.014	10.072 ± 0.084
rrBLUP	0.695 ± 0.014	0.481 ± 0.018	7.979 ± 0.142

The values represent the average performance across 15 repetitions of cross-validation, along with their standard deviations.

The best model is highlighted in bold.

**Figure 4:**
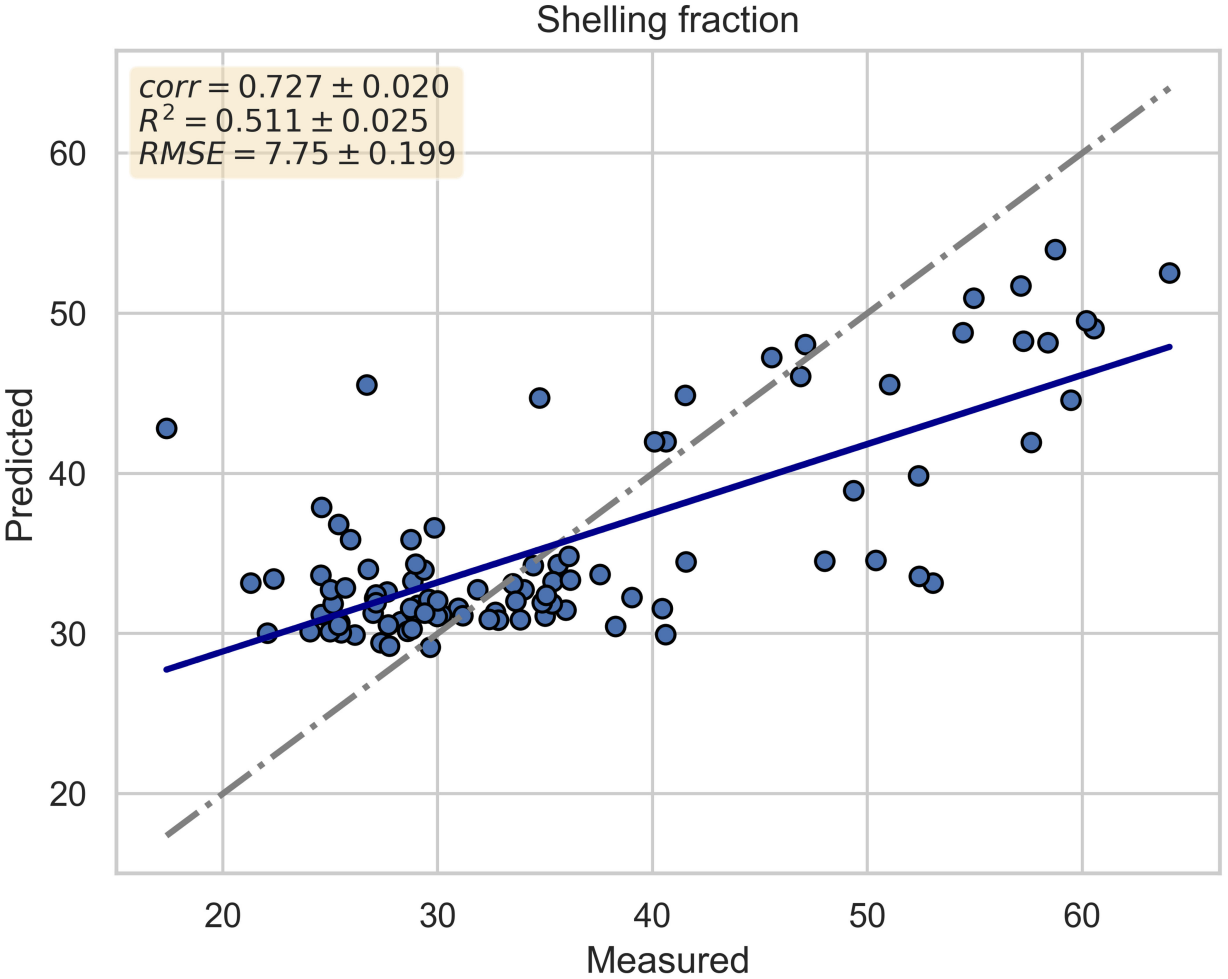
Scatter plot of cross-validated prediction results for the best model (Random Forest). The x-axis represents the actual shelling fraction values, while the y-axis represents the predicted values. The gray line represents the bisector, and the blue line represents the regression line.

**Figure 4** depicts the scatter plot of the cross-validated prediction results of the best model (Random Forest). The x-axis represents the actual shelling fraction values, while the y-axis represents the predicted values. Additionally, two lines are plotted to highlight the regression results: the gray line represents the bisector, and the blue line represents the regression line.

**Figure 5:**
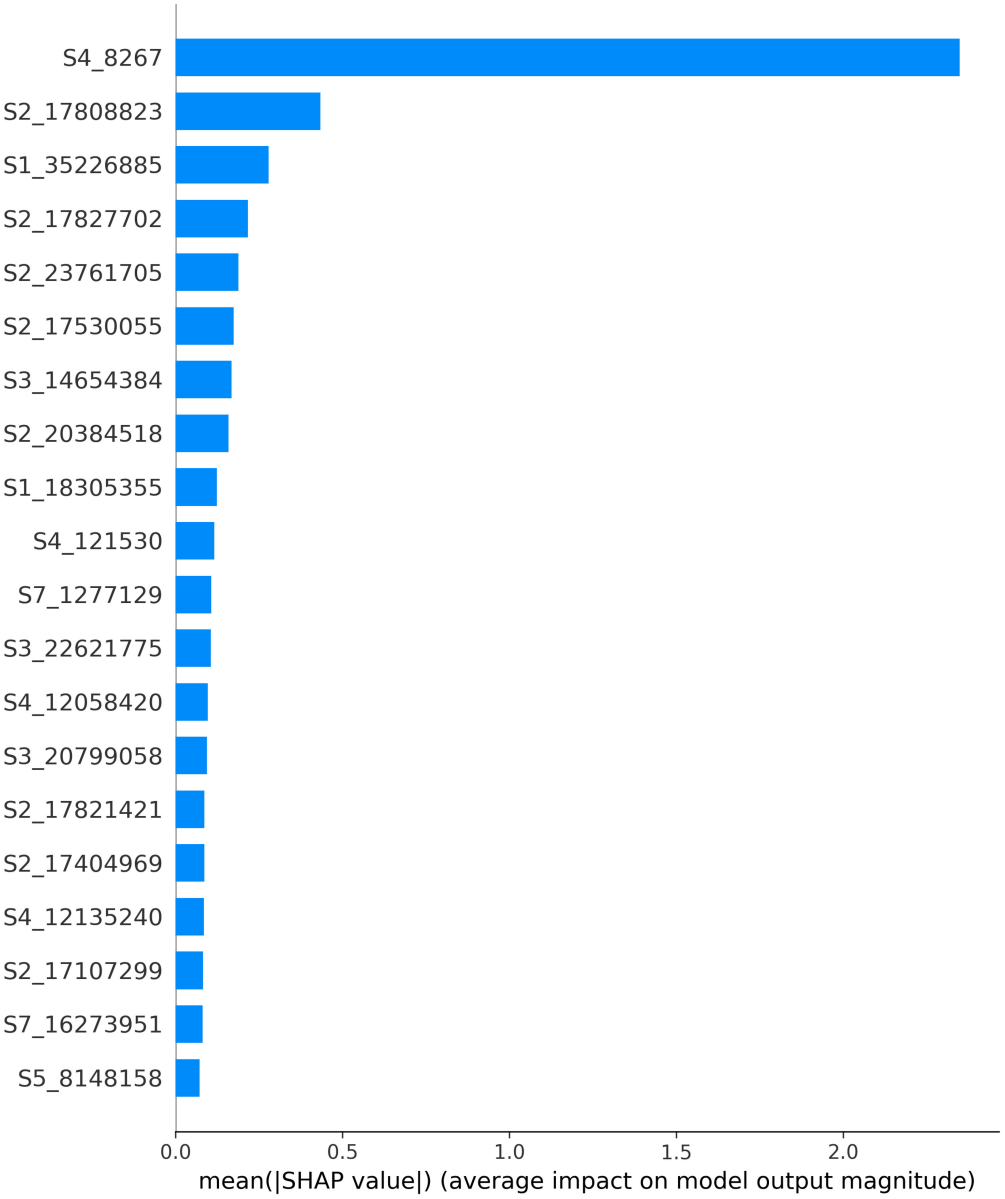
Feature importance calculated using SHAP values. The variables are ordered by importance, with the 20 most important variables depicted from most to least important based on the mean absolute value of the SHAP values. These values represent the average impact on the model output magnitude.

After evaluating the performance, SHAP values were computed. **Figure 5** illustrates the feature importance calculated using SHAP values. The variables are ordered by importance, with the 20 most important variables depicted from most to least important based on the mean absolute value of the SHAP values. These values represent the average impact on the model output magnitude. It’s worth noting that the feature importance visualized in **Figure 5** is for the features common to all folds post-feature selection. Therefore, for explainability, only the most stable features, those selected in every fold of cross-validation, were considered.

By far, the SNP “S4_8267”, located at the beginning of chromosome 4, was associated with the highest feature importance. Using available annotation for the Lauranne, genome, this SNP was found to reside within the gene Prudu_010622, predicted to encode a member of the plant QWRF motif-containing protein family. Protein BLAST revealed similarity with the Arabidopsis endosperm defective protein 1, previously shown to be essential for seed development Pignocchi et al. (2009).

## 4 Discussion

In this research, we developed an artificial intelligence workflow to apply eXplainable Artificial Intelligence (XAI) principles to tree-based machine learning models (Random Forests, Adaboost, and Gradient Boosting) for genomic prediction using almond germplasm data. The primary focus was on predicting phenotypic traits from SNP values obtained from 98 almond cultivars, aiming to study the correlation between genotype and phenotype and to provide interpretability for the predictions through SHAP values. Predicting shelling fraction from genomic data carries significant implications for both plant science and agricultural practices. Understanding the genetic foundations of shelling fraction is very important for the breeding programs, in order to predict the efficiency of the tree in producing seeds Sun et al. (2019); Upadhyaya et al. (2010). By leveraging genomic information, breeders can identify and select plants with desirable traits, accelerating the process of crop improvement. Furthermore, precise yield predictions enable farmers to optimize resource allocation, improve crop management practices, and mitigate risks associated with environmental variability and climate change Van Klompenburg et al. (2020); Alhnaity et al. (2019); Pant et al. (2021).

Our results have shown that the phenotypic variable, shelling fraction, is correlated with SNPs, as evidenced by a coefficient of determination of 0.511 ± 0.025. The *R* ^2^ value of 0.511 indicates that our model explains about 51.1% of the variance in the phenotypic trait based on genetic data alone. This level of accuracy is significant in the context of genomic prediction, where multiple factors influence phenotypic traits.

Machine learning models have proven effective in predicting this trait. In our study, we compared the machine learning models to benchmark models (gBLUP and rrBLUP), and the results demonstrated the added value of incorporating feature selection and advanced machine learning techniques in genomic prediction.

One of the strengths of this study is the utilization of an eXplainable Artificial Intelligence (XAI) framework. By employing SHAP values, which are model-agnostic, we were able to estimate the importance of SNPs in predicting phenotypic variation. Notably, we found that the SNP locus associated with the highest importance resides in a gene, Prudu 010622, showing high level of homology with Arabidopsis endosperm defective 1, previously implicated in seed development Pignocchi et al. (2009). Further functional studies might test whether Prudu 010622 is also playing a role in kernel development, thus affecting kernel yield.

### 4.1 Caveats and future perspectives

While this study demonstrates the potential of combining XAI with advanced machine learning models for genotype-to-phenotype predictions, there are several caveats and limitations to consider. Firstly, the dataset used in this study is relatively small, consisting of only 98 almond cultivars. This limited sample size may affect the generalizability and robustness of the findings. Future studies should aim to include larger and more diverse datasets to validate the results and improve the model’s predictive performance. Additionally, while our study focused primarily on genetic data, we acknowledge that incorporating environmental variables alongside genetic information holds promise for further improving prediction accuracy. Environmental factors, such as temperature, precipitation, soil composition, and management practices, play a crucial role in shaping crop yields Rebetzke et al. (2012). The absence of environmental data in our current models could be seen as a limitation of this study. Integrating environmental data into machine learning models can provide a more comprehensive understanding of the genotype-environment-phenotype interactions, leading to more accurate predictions and tailored agricultural interventions Gagneur et al. (2013); Guo and Li (2023); Barros and Offenbacher (2009). Future work should aim to combine genetic data with relevant environmental variables to enhance the robustness and applicability of genomic prediction models.

Validation steps are crucial for the broader application of these models in real-world breeding programs. Independent validation using external datasets should be performed to ensure the reliability and reproducibility of the findings. Moreover, functional validation of the identified SNPs and genomic regions is necessary to confirm their biological relevance and potential utility in breeding applications. Future research should also explore the application of these models to other crop species and phenotypic traits, expanding the scope of genotype-to-phenotype predictions in plant breeding.

## 5 Conclusions

The analysis presented in this study underscores the model’s predictive capacity, revealing a significant correlation between genotypes and shelling fraction across 98 almond cultivars. Our best ML model achieved an *R* ^2^ of 0.511 ± 0.025, outperforming traditional GS methodologies like gBLUP and rrBLUP. In addition, the application of XAI highlighted specific chromosomal regions and SNP positions of major importance in predicting the target phenotype, offering valuable insights for further genetic studies and contributing to more conclusive results. These findings emphasize the potential of integrating machine learning models with explainable AI to enhance the interpretability and accuracy of genomic predictions, ultimately advancing the field of plant breeding.

Furthermore, this study paves the way for future research endeavors exploring similar associations in other cultivar types and various phenotypic traits. By expanding the scope of investigation, we can deepen our understanding of genotype-phenotype relationships in plant science, ultimately contributing to advancements in agricultural practices and crop optimization.

## Data Availability

The original contributions presented in the study are included in the article/**Supplementary Material**, further inquiries can be directed to the author SP, stefano.pavan@uniba.it.
