# Supplementary material for: Explainable artificial intelligence for genotype-to-phenotype prediction in plant breeding: a case study with a dataset from an almond germplasm collection
Source: Front Plant Sci. 2024 Sep 9;15:1434229. doi: 10.3389/fpls.2024.1434229 (PMC11420924; doi:10.3389/fpls.2024.1434229)
Supplement: Supplementary file 1 [file DataSheet1.pdf]

Supplementary Material

1 SUPPLEMENTARY TABLES AND FIGURES

Table S1: List of 98 almond cultivars included in the study, along with their origin and values of the phenotypic variable (shelling fraction) for each year.

CODE	NAME	ORIGIN	Shelling fraction 2006	Shelling fraction 2007	Shelling fraction 2009	Shelling fraction 2012
-9-LEG-M-13	A grappolo	Italy	27,4058837	25,9960298	27,63485	26,89655
-9-LEG-M-130	Ai	France	41,0900154	43,6007025	41,09589	40,33194
-9-LEG-M-27	Albanese	Italy	?	29,8355521	27,83688	?
-9-LEG-M-95	Antonio de Vito	Italy	26,0401722	28,5467128	27,62078	26,26728
-9-LEG-M-64-3	Ardechoise	France	59,1628825	61,3021797	57,12188	52,89474
-9-LEG-M-112	Banchiere (Identity group 7)	Italy	26,5124275	27,6994025	33,22231	25,7037
-9-LEG-M-59	Bartre	Spain	13,8231982	21,4136192	17,83181	16,41205
-9-LEG-M-135	Bilarde	Italy	?	?	?	24,05272
-9-LEG-M-33	Burbank	U.S.	45,7755359	45,9568733	48,30028	42,12598
-9-LEG-M-87	Caporusso	Italy	30,3195635	31,013306	31,04541	25,18955
-9-LEG-M-109	Caputo	Italy	29,539801	29,2397661	30,61564	29,20863
-9-LEG-M-66	Catalini	Italy	30,0458716	37,2270742	33,83234	28,47011
-9-LEG-M-110	Catucedda (Identity group 10)	Italy	?	39,1107078	37,68889	35,8371
-9-LEG-M-88	Cavaliera	Italy	48,4010019	53,5184806	47,89392	42,20779

Continued on next page

Table S1: List of 98 almond cultivars included in the study, along with their origin and values of the phenotypic variable (shelling fraction) for each year. (Continued)

-9-LEG-M-96	Centopezze	Italy	31,646306	37,2866128	38,31418	32,95848
-9-LEG-M-72	Ciavea	Italy	30,82542	37,3480663	34,44329	31,36915
-9-LEG-M-17-2	Cristomorto (Identity group 9)	Italy	27,4310595	32,2557471	28,53717	26,13032
-9-LEG-M-116	D'Aloia	Italy	34,9283062	42,3093906	28,80184	37,82654
-9-LEG-M-49	Della Madonna di San Giovanni Rotondo	Italy	26,9005848	24,24	25,2541	21,86667
-9-LEG-M-83	Della madonna di Molfetta	Italy	24,4843603	24,6045244	26,36148	24,5
-9-LEG-M-7	Davey	U.S.	64,7736782	54,1976859	57,40741	52,64281
-9-LEG-M-134	Dehn	U.S.	65,32391	62,1137113	67,04545	61,79085
-9-LEG-M-39-2	Desmayo Largueta	Spain	28,111895	30,0299323	25,58286	23,32248
-9-LEG-M-67	Desmayo Rojo	Spain	24,9707766	27,1343829	23,35907	22,77946
-9-LEG-M-82	Dorée	France	27,0717142	30,6900103	30,60719	26,52632
-9-LEG-M-126	Drake	U.S.	41,0094637	40,0996264	43,90602	35,32551
-9-LEG-M-108	Falsa Catuccia	Italy	36,8493151	34,6892655	36,57205	34,29688
-9-LEG-M-155	Ferragnès	France	35,2983465	36,2126246	36,04207	30,20654
-9-LEG-M-119	Ferrante	Italy	31,7493113	28,3430233	31,79238	24,03101
-9-LEG-M-18	Ficarazza	Italy	41,3021363	43,1850789	43,59806	34,34191
-9-LEG-M-32-2	Filippo Ceo	Italy	35,7015469	34,3452825	37,60406	33,6712
-9-LEG-M-45	Flots	France	23,1657871	24,7241749	27,89598	22,57716

Continued on next page

Table S1: List of 98 almond cultivars included in the study, along with their origin and values of the phenotypic variable (shelling fraction) for each year. (Continued)

-9-LEG-M-10	Fourcouronne	France	21,3579433	24,112426	20,7502	?
-9-LEG-M-47	Fournat de Brézenaud	France	47,8834845	44,5081967	46,9592	48,22835
-9-LEG-M-3	Fragiulietta (Identity group 3)	Italy	32,382763	30,2544769	32,77487	29,22574
-9-LEG-M-106	Franciscudda	Italy	30,0609343	27,5140788	31,54085	27,19704
-9-LEG-M-122	Galgano	Italy	25,7768721	24,6870171	26,84515	24,69237
-9-LEG-M-42	Genco Laera	Italy	33,9578454	33,1843575	33,22476	30,95668
-9-LEG-M-121	Giunco di cozze di Alberobello	Italy	28,9317507	28,2153539	30,29638	28,08552
-9-LEG-M-85	Irene Lazolla	Italy	24,7928617	27,145359	27,15765	22,4208
-9-LEG-M-19	Lorena Tribuzio	Italy	29,8632219	28,443449	33,10811	23,81371
-9-LEG-M-139	Malagueña	Spain	54,6415981	47,632312	53,48542	48,4
-9-LEG-M-20	Mancina	Italy	25,2113759	31,2707182	37,72049	26,49728
-9-LEG-M-113	Marchione (Identity group 2)	Italy	30,4109589	30,1289566	33,47763	24,28884
-9-LEG-M-94-2	Marcona	Spain	22,4274496	28,2546368	26,83791	23,98524
-9-LEG-M-78	Maria Carolina Tribuzio	Italy	27,0928463	32,3890463	30,49155	?
-9-LEG-M-120	Maria Tribuzio	Italy	29,0607735	30,4659498	27,9148	23,53967
-9-LEG-M-34	Merced	U.S.	56,0902256	66,3000104	62,23507	57,57576
-9-LEG-M-114	Mincacetta	Italy	31,2693498	30,0129366	33,78545	28,79915
-9-LEG-M-74	Mincone	Italy	30,740276	31,4028314	?	27,76753

Continued on next page

Table S1: List of 98 almond cultivars included in the study, along with their origin and values of the phenotypic variable (shelling fraction) for each year. (Continued)

-9-LEG-M-84	Mollar de Taragona	Spain	42,617488	43,5661765	40,56761	39,48339
-9-LEG-M-79	Mollese di Canneto	Italy	56,5882353	53,2883642	52,49597	47,2561
-9-LEG-M-117	Pettolecchia (Identity group 4)	Italy	29,7567062	29,620563	30,7554	29,20892
-9-LEG-M-98	Montrone	Italy	25,4518061	26,5897205	31,25683	25,19737
-9-LEG-M-51	Ne plus ultra	U.S.	57,1167549	66,6283525	60,2349	56,78213
-9-LEG-M-70	Nikitski	Ukraine	58,3141668	58,5515298	57,78098	45,13783
-9-LEG-M-76	Nocella	Italy	21,5845048	24,297879	22,79608	20,77586
-9-LEG-M-103	Occhio Rosso di Trani	Italy	30,8622079	26,2969589	28,32278	25,08651
-9-LEG-M-62	Peerlees (Identity group 11)	Other	34,7062438	34,8001152	37,52784	31,93051
-9-LEG-M-36	Piangente	Italy	25,7309942	28,559322	29,28177	24,66144
-9-LEG-M-23	Picantili	Ukraine	57,5709779	55,5391865	47,74976	51,32027
-9-LEG-M-102	Pignatidde tardiva (Identity group 8)	Italy	30,785124	47,4065139	32,2449	29,56259
-9-LEG-M-77	Piscalze	Italy	28,5981308	28,9791438	29,05569	24,15414
-9-LEG-M-57	Pizzuta d'Avola	Italy	26,4119601	26,7093297	25,32258	25,32691
-9-LEG-M-6	Primicerio	Italy	27,4022989	28,7128713	30,56042	28,30189
-9-LEG-M-181	Primorsky	Ukraine	69,2748092	69,4779116	24,62312	?
-9-LEG-M-26	Pulita	Italy	20,7854406	22,4902216	22,76364	19,14591
-9-LEG-M-91	Putignano	Italy	23,6495389	27,4934952	22,70463	26,17398

Continued on next page

Table S1: List of 98 almond cultivars included in the study, along with their origin and values of the phenotypic variable (shelling fraction) for each year. (Continued)

-9-LEG-M-24	Rabasse	France	30,6976744	33,4065934	38,76652	27,87685
-9-LEG-M-92	Rachele	Italy	24,4853235	24,8646958	27,89768	25,53191
-9-LEG-M-38	Rachele tenera	Italy	39,8109244	34,6638655	37,63676	32,60644
-9-LEG-M-104	Rachelina	Italy	24,9710313	29,7029703	31,89655	18,02253
-9-LEG-M-28	Rana gentile (Identity group 6)	Italy	36,9218501	32,7649208	37,78234	33,97746
-9-LEG-M-90	Reale	Italy	33,7792642	35,4983203	34,43344	30,82524
-9-LEG-M-56	Retsou	Greece	55,6208376	48,0666822	54,98339	50,81433
-9-LEG-M-137	Ridenhome (Identity group 5)	U.S.	78,3420407	52,8026086	57,43671	46,41694
-9-LEG-M-118	Riviello	Italy	34,9542567	37,7867028	37,39726	34,29711
-9-LEG-M-44	Rossa	Italy	27,1545828	32,122213	30,58054	27,56211
-9-LEG-M-73	Santeramo	Italy	50,4024145	50,0044078	54,69543	46,45892
-9-LEG-M-93	Santoro	Italy	33,9607843	38,1128585	34,60601	32,72562
-9-LEG-M-35	Scorza verde	Italy	34,978355	31,5789474	34,49001	26,3877
-9-LEG-M-58	Senz’arte	Italy	25,5395683	25,3697383	28,28947	22,52874
-9-LEG-M-14	Sultane	France	46,7181467	48,3771252	49,74425	43,59949
-9-LEG-M-69	Summetrike	Greece	44,1624365	62,2958654	66,10801	61,0654
-9-LEG-M-15	Tardy nonpareil	U.S.	61,0375276	57,6388889	64,35331	54,86862
-9-LEG-M-41	Tenente	Italy	27,71261	32,9637097	32,09581	25,96401

Continued on next page

Table S1: List of 98 almond cultivars included in the study, along with their origin and values of the phenotypic variable (shelling fraction) for each year. (Continued)

-9-LEG-M-43	Texas	U.S.	48,7596678	52,5348366	46,27995	49,89171
-9-LEG-M-63	Tondina	Italy	23,4865062	27,6353276	25,02317	24,34626
-9-LEG-M-89	Tournefort	France	26,4661654	27,7996071	28,32103	24,21746
-9-LEG-M-55	Tribuzio	Italy	29,4880104	30,1215278	32,22037	43,51051
-9-LEG-M-68	Chino (Identity group 1)	Italy	40,3594535	40,2199356	42,19949	33,33333
-9-LEG-M-133	Vesta	U.S.	60,8433735	56,6787004	57,31922	53,75
-9-LEG-M-80	Viscardia	Italy	44,5719489	39,0730346	44,91701	33,21751
-9-LEG-M-86	Vuoi o non vuoi	Italy	28,3697953	?	29,79233	28,78261
-9-LEG-M-11	Yaltinskyi	Ukraine	39,5365677	45,1190476	42,26328	35,59738
-9-LEG-M-105	Zanzanidde	Italy	28,0620155	29,7066015	27,94702	23,65805
-9-LEG-M-53	Zia comara	Italy	36,5950676	38,7966805	44,36702	33,35855
-9-LEG-M-71	Zin zin	Italy	33,6270191	37,1819961	33,88773	31,3783

2 SOFTWARE INFORMATION

The analysis has been tested on the following system:

- macOS Sonoma 14.5

The analysis was primarily conducted in Python, with the implementation of the gBLUP and rrBLUP models carried out in R. Software versions used:

- Python: 3.12.4
- R: 4.4.0

Version Numbers for Python Packages in the macOS environment:

- numpy version: 1.26.4
- pandas version: 2.2.2

- matplotlib version: 3.8.4
- shap version: 0.46.0
- sklearn version: 1.5.1
- xgboost version: 2.1.0
- seaborn version: 0.11.2
- scipy version: 1.13.1
- statannotations version: 0.5.0

Version Numbers for R Packages in the macOS environment:

- rrBLUP version: 4.6.3
- Metrics: 0.1.4
